# ‘You can’t just put somebody in a situation with no armour’. An ethnographic exploration of the training and support needs of homecare workers caring for people living with dementia

**DOI:** 10.1177/14713012211023676

**Published:** 2021-06-10

**Authors:** Monica Leverton, Alexandra Burton, Jules Beresford-Dent, Penny Rapaport, Jill Manthorpe, Hassan Mansour, Stefanny Guerra Ceballos, Murna Downs, Quincy Samus, Briony Dow, Kathryn Lord, Claudia Cooper

**Affiliations:** Division of Psychiatry, 4919 University College London, London, UK; Centre for Applied Dementia Studies, 1905 University of Bradford, Bradford, UK; Division of Psychiatry, University College London, London, UK; NIHR Policy Research Unit on Health and Social Care Workforce, 4616 King’s College London, London, UK; Division of Psychiatry, 4616 University College London, London, UK; Centre for Applied Dementia Studies, 1905 University of Bradford, Bradford, UK; Department of Psychiatry and Behavioral Sciences, Johns Hopkins Bayview, 1466 Johns Hopkins University, Baltimore, MD, USA; National Ageing Research Institute, Parkville, VIC, Australia; 2281 University of Melbourne, Parkville, VIC, Australia; Deakin University, Waurn Ponds, VIC, Australia; Centre for Applied Dementia Studies, 1905 University of Bradford, Bradford, UK; Division of Psychiatry, 1905 University College London, London, UK

**Keywords:** dementia care, dementia training, domiciliary care, qualitative research methods, ethnography

## Abstract

**Background:**

Homecare workers carry out complex work with people living with dementia, while under-supported, undervalued and undertrained. In this ethnographic study, we explore the skills, training and support needs of homecare workers supporting people living with dementia.

**Research Design and Methods:**

We conducted 82 interviews with people living with dementia (*n* = 11), family caregivers (*n* = 22), homecare staff (*n* = 30) and health and social care professionals (*n* = 19) and conducted 100-hours of participant observations with homecare workers (*n* = 16). We triangulated interview and observational findings and analysed data thematically.

**Results:**

We developed four themes: 1) ‘Navigating the homecare identity and role’: describing challenges of moving between different role identities and managing associated expectations, 2) ‘Developing and utilising relational and emotional skills’: boundaries between caring and getting emotionally involved felt blurred and difficult to manage, 3) ‘Managing clients who resist care’: homecare workers experienced clients’ reactions as challenging and felt “thrown to the wolves” without sufficient training, and 4) ‘Drawing on agency and team support’: homecare work could be isolating, with no shared workplace, busy schedules and limited opportunity for peer support.

**Discussion and Implications:**

It is important that training and support for homecare workers addresses the relational, emotional and rights-based aspects of the role. Where a flexible, responsive, person-centred service is required, corresponding training and support is needed, alongside organisational practices, taking account of the broader context of the homecare sector.

## Background and objectives

There are 850,000 people living with dementia in the United Kingdom (Wittenberg et al., 2020) and over 46 million people globally (Prince et al., 2015). Over 680,000 paid care workers in England provide direct care to people in their own homes (Skills for Care, 2018). Two-thirds of these workers, termed homecare workers, direct care workers or domiciliary support workers regularly care for people living with dementia (Carter, 2016). The homecare sector is in increasingly high demand and will grow substantially as society ages and care shifts further to domiciliary settings, where most people with dementia prefer to remain living.

Over 9000 Care Quality Commission (CQC) regulated homecare providers operated across England in 2018 (Skills for Care, 2020). The vast majority are independent or voluntary organisations (UKHCA, 2016). Homecare workers are predominantly female (84%), white (76%) and British (83%), with an average age of 43 years (Skills for Care, 2019), similar to the wider adult social care workforce. Most work part-time and 56% are employed on zero-hour contracts (Skills for Care, 2020). They form the largest proportion of paid staff in the home setting, delivering personal care, assistance with domestic activities, basic nursing care and companionship.

Homecare workers are the ‘front line’ of the social care system (The King’s Fund, 2018). However, the sector faces challenges including poor recruitment, retention, low pay and morale (The King’s Fund, 2018). Homecare workers receive limited to no supervision or training, the quality of their work is often publicly scrutinised and their value to clients living with dementia and their family members may be unrecognised.

Mandatory training was introduced as the Care Certificate in England, in 2015. Social care staff are expected to complete the Care Certificate during their induction (Skills for Care, 2015); however, it has not been taken up universally (Skills for Care, 2018). This may be related to the fact that it is not an accredited national qualification and employers are not obliged to offer it to staff. The Care Certificate contains minimal awareness training in relation to dementia, and there is currently no requirement for homecare workers to complete dementia-specific training; a third of English homecare workers have not received dementia training (Skills for Care, 2018). More dementia-specific training could improve homecare provision and in turn, it may improve client quality of life, reduce distressing behaviours (Surr et al., 2017) and subsequently may be cost-effective in supporting people with dementia to live at home for longer (Cooper et al., 2017). Developing such training requires the perspectives of all stakeholders involved in homecare provision, including clients living with dementia.

Ethnographic research methods are well-suited to exploring care at home (Briggs et al., 2003), particularly with people living with dementia (Leverton et al., 2019; MacLaren et al., 2017). Participant observations can capture the perspectives of those with more severe dementia, who may be unable to express verbally (Mansell, 2011). In the ‘Broadening our Understanding of Good Home Care’ (BOUGH) programme, researchers ethnographically explored the experiences of homecare workers supporting people living with dementia, from one commercial UK agency (Pollock et al., 2020; Schneider et al., 2019). Preserving and maintaining clients’ physical, mental, emotional and social well-being was of paramount importance. Receiving recognition and feeling able to make even small improvements in clients’ well-being were seen as part of the ‘implicit moral balance’ in homecare, highlighting the potential for reward and recognition to improve staff retention (Schneider et al., 2019). The authors emphasised the value of social skills; family carers prioritised homecare workers as companions for their relatives with dementia, beyond practical care tasks (Pollock et al., 2020). Therefore, understanding how to provide a holistic care approach with empathy for clients’ preferences and social needs could potentially be developed through training, alongside support that enables homecare workers to feel valued in their role.

In the current study, we extended the existing evidence-base in a large ethnographic study encompassing several homecare agencies, including the perspective of clients living with dementia. Our methods provide a complementary perspective to the BOUGH study (Schneider et al., 2019), using researcher-observers to directly observe the practice of homecare workers. We took an ethnographic approach to explore our research question across perspectives of key stakeholders: what are the skills, training and support needs of homecare workers providing care for people living with dementia? This study informed the development of a coproduced training and support intervention for homecare workers in a wider programme of work (Lord et al., 2020).

## Methods

### Study design

We conducted a multi-site study of homecare for people living with dementia using an ethnographic approach. We held qualitative semi-structured interviews between April and August 2018 with people living with dementia, family carers, health and social care professionals and homecare staff. We undertook participant observations with homecare workers providing care to clients living with dementia between September 2018 and March 2019. Participant observation methods were informed by our earlier review of homecare observational studies (Box 1). We took a rapid ethnographic approach, prioritising breadth over depth (Utarini et al., 2001; Vindrola-Padros & Vindrola-Padros, 2018). We observed care in several agencies for a shorter time duration, as opposed to more intensive observations in one agency. This approach allows for greater generalisability and faster translation of research into practice (Knoblauch, 2005). As this study informed intervention development for the homecare sector, it was important to have a broad understanding across homecare agencies and stakeholder groups.

**Box 1.** Methodological design considerations for conducting participant observations in the home setting.

Observation characteristics (Leverton et al., 2019)	*Applied to our study design*
Terminology	*Participant observation*
Structure	*Guided observation*
Data collection	*Naturalistic (in person without researcher intervention) + ethno-interview technique*
Number of researchers; researcher role	*3* ‘*researcher-observers*’*, each observing care provided by 2 homecare agencies; conversational only (no participation in care tasks)*
Rapport building	*1–2 familiarisation visits (contextual notes only)*
Time spent observing	*Pre-decided: 1–2 familiarisation visits + up to 5 observation visits; duration per observation determined by client’s visit time (capped at 2 hours to prevent researcher fatigue and observation saturation)*
Recording data	*Fieldnote* ‘*jottings*’ *using pen and paper during visit, full notes typed up no more than 48-hours later where possible (to prevent limitations of memory); reflective stance recorded alongside fieldnotes and in reflective journal*
Validation and triangulation	*Data triangulated with interviews, weekly team discussions, fieldnotes read by wider research team and double-coded during analysis,* ‘*fair dealing*’ *(**Mays & Pope, 2000**)*
Ethical considerations	*Researcher-observers assessed capacity of clients with dementia at the start of observations and at each visit; regularly informing clients of purpose for visit*

### Setting and sample

#### Qualitative interviews

We recruited key stakeholders involved in homecare provision for people living with dementia. This included people diagnosed with dementia with capacity to consent and carers (relatives or friends) from three National Health Service (NHS) memory services, a private homecare agency, an Alzheimer’s Society Experts by Experience group and Twitter. Health and social care professionals who were involved in commissioning or planning homecare for people living with dementia were recruited through UCL, four NHS memory services and one local authority. We recruited homecare staff from ten urban and semi-rural/rural homecare agencies, who worked with local authority and/or privately funded clients with dementia. Homecare staff included agency managers, office support staff and direct homecare workers. All gave written informed consent.

Participants were purposively recruited to include a range of ages, ethnicities, roles (homecare staff, health and social care professionals), relationship to the person with dementia (family carers), experience with homecare services (people living with dementia) and shift-pattern and client-type (homecare workers).

#### Participant observations

We purposively sampled homecare agencies for diversity of location (in urban or rural/semi-rural locations), CQC (regulator) rating and care provision (i.e. agencies with 15-minute visits and agencies with a one-hour minimum visit policy). We contacted agency managers to seek their agency’s participation. Of the 11 homecare agencies participating in qualitative interviews, we purposively approached seven varied agencies to also take part in observations. Of these, one manager declined and another consented, but we lost contact with the agency after the manager moved. We approached three additional agencies that were not involved in the interviews. Of these, one manager agreed their agency could participate, and two other agency managers expressed preliminary interest that was not sustained.

Homecare agency managers were asked to complete a questionnaire about their agency and to direct us to homecare workers who supported client(s) living with dementia. We excluded staff intending to leave the agency within 6 months. We provided a participant information sheet, sought written consent and demographic information. Homecare workers and managers then invited clients diagnosed with dementia and their family members to participate. A nominated consultee (i.e. a family member) provided written consent if the person living with dementia lacked capacity. We obtained written consent from family members, healthcare professionals and other homecare workers who were present during observation visits to include their interactions in fieldnotes. We purposively recruited homecare worker and client dyads to include diverse visit types (i.e. personal care and respite visits), schedules and durations.

### Data collection

**Figure 1.**
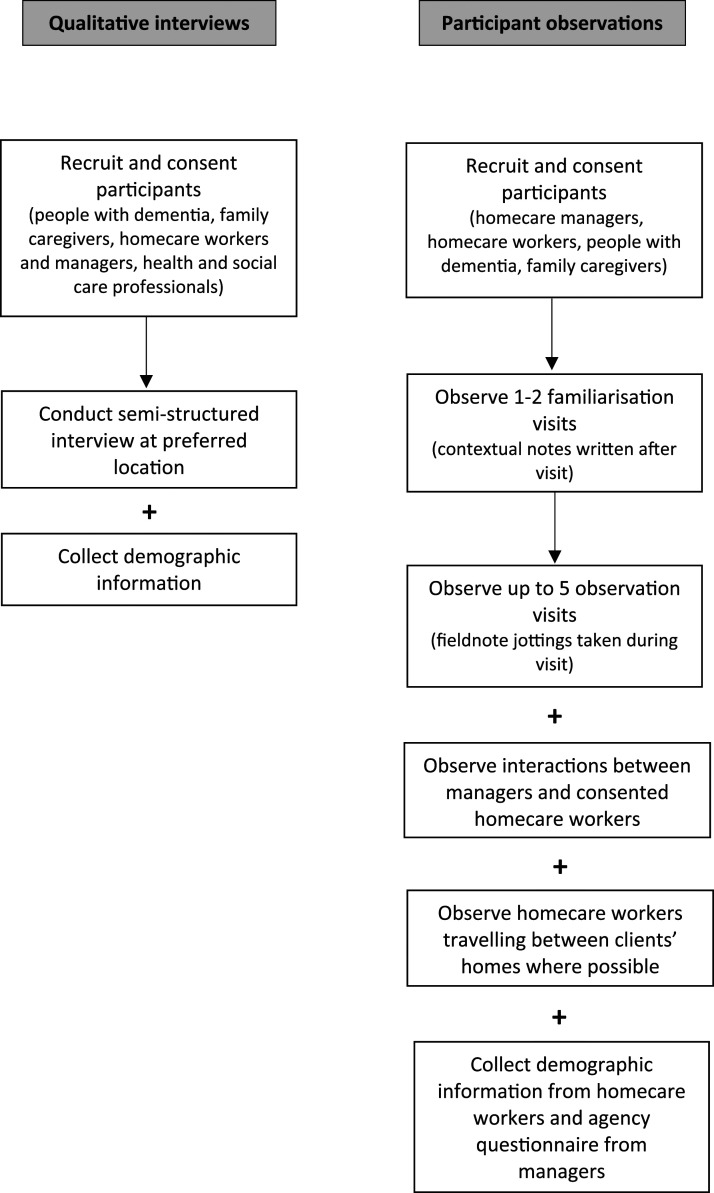
Mapping the data collection process.

The data collection process is displayed in Figure 1.

#### Qualitative interviews

We conducted interviews at locations convenient for participants and interviewed people living with dementia and family carer dyads together if preferred. Participants were offered a £20 voucher for their time. Interviews lasted around 1 hour, were audio-recorded and followed a semi-structured topic guide (Supplementary Material). Questions focused on what a training and support programme for homecare workers supporting clients living with dementia should comprise and how it should be delivered. We ceased interviews when data saturation was reached, gathering data until the point of ‘diminishing returns, when nothing new is being added’ (Bowen, 2008). This was decided upon by reflecting on and reading earlier interview transcripts and meeting with data-collecting co-authors to iteratively discuss preliminary patterns and codes.

#### Participant observations

Three non-clinical researcher-observers from psychology and sociology backgrounds travelled with and observed homecare workers with clients living with dementia. Observations included homecare workers providing care, including personal care, and interpersonal interactions during home visits and in agency offices. For each client, we conducted up to two familiarisation visits, during which researcher-observers did not take fieldnotes, aiming to foster familiarity and build rapport (MacLaren et al., 2017); they made contextual notes after the visit. Up to five further observation visits were made during which researcher-observers took ‘jottings’ (brief fieldnotes) (Emerson et al., 2011).

A semi-structured observation guide (Supplementary Material) focused the observations, aided consistency between researcher-observers and prompted reflexivity; researcher-observers kept reflective journals alongside fieldnotes. The description by Adler and Adler (1987) of a ‘peripheral’ observer aligns well with the observer stance adopted: we gained first-hand insider perspectives without functionally participating in the homecare. Researcher-observers interacted conversationally as felt natural and used an ethno-interviewing technique (unstructured naturalistic conversation) to enrich fieldnotes (DeWalt & DeWalt, 2011).

Researcher-observers viewed personal care only with consent from the person living with dementia or their nominated consultee (under England and Wales’ Mental Capacity Act 2005) and checked for signs of distress from the person living with dementia.

### Data analysis

We conducted a reflexive thematic analysis (Braun & Clarke, 2006, 2020), triangulating interview and observation data. Both data sources were transcribed verbatim, and identifiable information was removed. ML read all data for familiarity. ML and co-authors inductively open and double-coded 25% of the interview transcripts across participant groups and 15% of the observation fieldnotes. Similar to the ‘Following a thread’ approach (Moran-Ellis et al., 2006), we explored how codes from one dataset followed into the other until we developed one interwoven coding framework. We then applied this framework to the remaining interview transcripts and half of the observation fieldnotes until no new codes were found (i.e. thematic saturation). The remaining fieldnotes were read in detail and compared against the framework to ensure verification, comprehension and completeness of the data (Morse et al., 2002). We refined and defined themes and explored divergences between interview and observation data.

We used anonymised identifiers for interview participants and pseudonyms when presenting supporting fieldnotes: ‘A’ names for homecare workers, ‘B’ for clients with dementia and ‘C’ for family carers.

## Findings

### Sample characteristics

We interviewed 82 participants: 11 people living with dementia, 22 family carers (including 3 dyads, for whom family carers were interviewed separately, though for 2 dyads, the family carer was present when the person living with dementia was interviewed), 19 health and social care professionals and 30 homecare staff (7 managers, 4 office staff and 19 homecare workers) (Table 1).

**Table 1.** Demographic information of interview participants (*n* = 82).

Characteristics	Category	Homecare managers and support staff, *n* (%) or *mean* (*SD*)	Homecare workers, *n* (%) or *mean* (*SD*)	People with dementia, *n* (%) or *mean* (*SD*)	Family carers, *n* (%) or *mean* (*SD*)	Health and social care professionals, *n* (%) or *mean* (*SD*)
*N*		11	19	11	22	19
Age		*47.3* (*9.5*)	*48.9* (*12.9*)	*78.6* (*7.8*)	*57.7* (*14.3*)	*41.4* (*10.9*)
Gender	Female	9 (81.8)	16 (84.2)	5 (45.5)	12 (54.5)	13 (68.4)
	Male	2 (18.2)	3 (15.8)	6 (54.5)	10 (45.5)	6 (31.6)
Ethnicity	White – British	7 (63.6)	15 (78.9)	8 (72.2)	9 (40.9)	9 (47.4)
	White – Irish	1 (9.0)	0 (0.0)	1 (9.1)	0 (0.0)	1 (5.3)
	White – other	0 (0.0)	1 (5.3)	0 (0.0)	0 (0.0)	4 (21.1)
	Asian – Indian	0 (0.0)	0 (0.0)	0 (0.0)	6 (27.3)	2 (10.5)
	Asian – Bangladeshi	0 (0.0)	0 (0.0)	0 (0.0)	4 (18.2)	0 (0.0)
	Black/Black British – African	2 (18.2)	1 (5.3)	0 (0.0)	0 (0.0)	0 (0.0)
	Black/Black British – Caribbean	1 (9.0)	1 (5.3)	0 (0.0)	0 (0.0)	0 (0.0)
	Other	0 (0.0)	1 (5.3)	2 (18.2)	3 (13.6)	3 (15.8)

We observed homecare provided by six commercial agencies (Table 2) with 16 homecare workers (Table 3) and 17 people living with dementia (Table 4). Four homecare workers took part in both the qualitative interviews and participant observations. Twenty homecare workers consented to be observed. Three dropped out prior to familiarisation visits (due to illness, client declining participation and overlap in the researcher-observer’s schedule, respectively). One homecare worker did not participate following one familiarisation visit, due to illness. Two people living with dementia from one agency consented to observations but did not take part (due to overlap with the researcher-observer’s schedule and reaching our purposive sampling target within that agency). Two people living with dementia were observed during familiarisation visits only, due to subsequent hospitalisation. We observed 104 homecare visits (including 24 familiarisation visits), recording 100-hours of observations, with additional observations within agencies’ offices and during travel.

**Table 2.** Characteristics of homecare agencies participating in observations (*n* = 6).

Homecare agency	Location	CQC rating	Total number of clients (% of caseload with dementia or memory problem)	Homecare workers on zero-hour contract (% of all employed)	Care funding	Homecare workers observed	Clients with dementia observed
1	London	Good	91 (39.5%)	85 (100.0%)	Private only	1	1
2	London	Good	28 (53.6%)	1 (6.6%)	Private only	2	2
3	South England	Good	150 (4.6%)	90 (100.0%)	Local authority only	3	2
4	South England	Requires improvement^a^	180 (45.0%)	67 (95.7%)	Private and local authority	5	7
5	North England	Outstanding	112 (62.5%)	74 (93.7%)	Private and local authority	3	3
6	North England	Good	196 (31.6%)	120 (95.2%)	Private and local authority	2	2

Note: CQC: Care Quality Commission.

^a^CQC rating changed from ‘Good’ to ‘Requires Improvement’ at the start of the observation period.

**Table 3.** Characteristics of homecare workers observed providing homecare (*n* = 16).

Characteristics	Category	*N* (%) or *mean* (*SD*)
Age^a^		*49* (*5*)
Ethnicity	White – British	12 (75)
	White – other	1 (6)
	Black/Black British – Caribbean	1 (6)
	Black/Black British – African	2 (13)
Contract type	Employed on zero-hour contract	5 (31)
Employment	Working part-time	5 (31)
	Working full-time	11 (69)
Years worked in care	6 months–1 year	3 (19)
	1–3 years	4 (25)
	3–5 years	1 (6)
	5–10 years	4 (25)
	10 years or more	4 (25)
Years worked in current agency^b^	Less than 6 months	1 (6)
	6 months–1 year	3 (18)
	1–3 years	7 (44)
	3–5 years	2 (13)
	5–10 years	2 (13)
Method of travel	Walk only	1 (6)
	Cycle	1 (6)
	Car	10 (62)
	Bus/bus with a walk	4 (25)
Personal experience of dementia	Yes	6 (38)
	No	10 (62)

^a^Three homecare workers chose not to disclose this.

^b^One homecare worker was unable to specify.

**Table 4.** Characteristics of people living with dementia observed receiving homecare (*n* = 17).

Pseudonym	Age	Sex	Living situation	Capacity to consent	Care funding	Length of each visit
Betty	Missing data	Female	Lives alone	Yes	Private	3 hours
Beverley	77	Female	Lives with spouse	No	Private	1 hour
Bonnie	84	Female	Lives alone	Yes	Private	1.5–3 hours
Belinda	82	Female	Lives alone	No	Local authority	30 minutes
Barbara	80	Female	Lives with son	No	Local authority	30–45 minutes
Brian	61	Male	Lives with spouse	No	Local authority	3 hours (sitting service)
Beth	85	Female	Lives with spouse	No	Local authority	15–30 minutes (4-hour respite visits twice weekly)
Beatrice	96	Female	Lives alone	No	Local authority	3-hour shifts within 24-hour care package
Brenda	93	Female	Lives alone	Yes	Local authority	30 minutes
Benji	84	Male	Lives with spouse	No	Local authority	30 minutes
Bernice	89	Female	Lives alone	Yes	Local authority	15 minutes
Bridgette	94	Female	Lives alone	Yes	Local authority	30 minutes
Boris	77	Male	Lives with spouse	No	Private	2 hours
Bara	98	Female	Lives alone	No	Private	1 hour
Benita	88	Female	Lives alone	No	Private	1–5 hours
Bryony	99	Female	Lives alone	No	Local authority	30 minutes
Bea	89	Female	Lives alone	No	Local authority	30 minutes

### Qualitative analysis findings

We describe four themes responding to our research aim: (1) ‘Navigating the homecare identity and role’, (2) ‘Developing and utilising relational and emotional skills’, (3) ‘Managing when clients resist care’ and (4) ‘Drawing on agency and team support’. All themes drew on both methods of data collection. While we did not have an a priori intention to give greater weight to either data source, the balance between them varied by the stakeholder group. Interviews were the primary source of data for non-frontline professionals (e.g. homecare managers) who were not usually present in observations and for family carers. Perspectives of clients living with dementia were gleaned mostly from observations, which allowed perspectives to be conveyed in-the-moment, circumventing memory loss. Health and social care professionals’ perspectives featured in both. Observations also allowed us to observe interactions occurring between participants. Interviews and observations seemed to contribute equally to our findings regarding homecare worker perspectives. All participant groups identified challenges of homecare for people living with dementia and plausible solutions to embed into homecare workers’ training and support. Some solutions were beyond the scope of training, requiring organisational changes, for example, to funding or scheduling arrangements.

## Navigating the homecare identity and role

Across stakeholders, there was ambiguity around the role of homecare workers, including boundaries and limits. We outline the different role identities held by homecare workers (subtheme 1) and the subsequent expectations and tensions that arose amongst stakeholders (subtheme 2).

### Subtheme 1: Role identity

Homecare workers often adopted parallel role identities, including a proxy-healthcare professional, friend to clients and their family members or the home-help. Holding and sometimes moving between identities could pose challenges.

#### Proxy-healthcare professional

The homecare role was described as needing the skills traditionally associated with health and social care professional roles: of counsellor, nurse or warden responsible for clients’ safety and well-being. We observed situations where homecare overlapped with healthcare visits, which often involved the healthcare professional positioning the homecare worker as their proxy to carry out tasks in their absence. For example, one homecare worker was instructed by a district nurse to care for a client’s wounds. In other examples, homecare workers reiterated healthcare professionals’ advice to encourage clients living with dementia to complete necessary tasks:

‘*Angela reminds Betty that the doctor has told her she must stop using soap to wash her private areas as the soap has been causing her infections. Betty agrees and repeats what she remembers of this correctly, and then drains the sink to remove the soap from the water.*’ *(Agency 1-Observation)*

Homecare workers were sometimes uncertain of the extent to which supporting clients with their health care fell within the remit of their role:

‘*Ashley asks Abbey if they should put in eye drops as Beverly’s eye looks sore. Abbey says that they cannot issue medication.*’ *(Agency 3-Observation)*

Some healthcare professionals and family carers felt homecare workers lacked sufficient training to carry out health-related tasks. Whereas homecare managers perceived that the value of homecare workers’ familiarity and understanding of clients, in addition to their training and experience, should situate them as valued contributors to the healthcare multidisciplinary team, although this was unrecognised and undervalued:

‘*…Yes, they are not as highly qualified as a district nurse, or an occupational therapist, but they have training, and they are in there seeing the client every day. Whereas nurses might be going in once a week… or when there’s a problem. But [their] opinions on things get thrown back quite often.*’ *(Homecare Manager 6-Interview)*

#### Personal companion

Homecare workers were valued by clients and family members as companions, friends or likened to a family member. Homecare workers in turn considered building positive relationships as essential to good care provision and a cherished part of the job, using terms such as ‘aunty’ or ‘good friend’ to describe their clients. Discourses around the homecare worker as a companion contrasted with professional discourses, giving rise to a different set of rules, relationships and interactions. They suggested a sense of reciprocity:

‘*Angela says her and Betty* “*work like each other’s brains*”*.*’ *(Agency 1-Observation)*

‘*Clara describes Alison as an* “*angel*” *and a* “*God-send*”*, saying that she is like a daughter to her and Benji. She tells me that Alison was the first homecare worker that Benji had and Alison had also just started being a homecare worker, so they learned what to do together.*’ *(Agency 4-Observation)*

The quote above implies that some skills are not taught but evolve throughout the course of the relationship with the client and their family.

With the identity of personal companion, professional boundaries could blur. In some cases, the sense of being part of the in-group led to homecare workers passing judgements on less involved or distanced family members, or becoming involved in activities beyond the remit of the homecare role:

‘*And then I went in the next day, [she] looked anxious again and I said,* “*what are you worrying about?*”*. She said,* “*I don’t want my children here when I write the will*”*. So, I had to ring the daughter and son and tell them that she wants me be there, and I actually witnessed her will.*’ *(Homecare Worker 12-interview)*

#### Home-help

Homecare workers also provided domestic support, substituting for aspects of life the client could no longer do:

‘*And you do have to do everything, from personal care to medication to gardening to cooking. You really are sort of being the eyes and the ears and the hands of that person.*’ *(Homecare Manager 3-interview)*

This identity was most clear cut for homecare workers as domestic tasks were outlined in the clients’ care plan. However, challenges arose when balancing these tasks with the relational and social needs of clients and family members.

### Subtheme 2: Role expectations

Role ambiguity gave rise to tensions. Homecare workers faced dilemmas when stakeholders had different expectations of their role. One person living with dementia expressed frustration when homecare workers seemed too focused on completing the care plan’s set tasks:

‘*…give more time for the people, rather than the doing… It then gives the [homecare worker] the time to ask the person how they are, instead of fussing around doing the practical things; they can actually talk to the person then.*’ *(Person living with dementia 17-interview)*

Balancing the care plan with the client and their family’s wishes and expectations tested homecare workers’ professional boundaries when asked to partake in tasks or activities beyond their role’s remit, or outside scheduled visit times. In one observation, a family carer asked a homecare worker to monitor the client via a camera, beyond her working hours:

‘*Angela shows me the motion-sensored camera in Betty’s bedroom that Cliff had asked her to monitor. The camera is connected to an app on Angela’s phone which she often checks to see that Betty is safe when she is alone. Angela says that if she saw anything was wrong, she would be the first to rush over.*’ *(Agency 1-Observation)*

Being asked to take on such a position could bring rewards of esteem and satisfaction from being trusted and autonomous, but also anxieties from onerous or intrusive expectations; Angela reported ‘not being able to switch off’ from her role, with other homecare workers describing a weight of responsibility. Some homecare workers were more cautious of the repercussions of taking on responsibility beyond their role:

‘*I get on well with her [the client’s wife], it’s just when she wants to have things her own way, and I know that it’s not the right way, rather… It’s the policies, the procedure that has to be followed… we have to abide by the rules.*’ *(Homecare Worker 13-Interview)*

However, homecare workers’ competency or value could be questioned if they did not meet stakeholders’ expectations; one pertinent example described the homecare role as ‘low level’ work (Healthcare Professional 18-Interview). Some homecare workers reported asking for advice or support from their manager to navigate such situations without disappointing stakeholders.

## Developing and utilising relational and emotional skills

Close and complex relationships often developed between homecare workers and clients living with dementia and their family carers. Homecare workers required key relational and emotional skills to develop (subtheme 1) and manage (subtheme 2) these relationships.

### Subtheme 1: Building relationships with clients living with dementia and their family carers

All stakeholders felt that ‘getting to know’ and becoming familiar with clients and their families were critical to homecare for people living with dementia. The skills required included a need to ‘*talk the same language*’ *(Homecare Worker 23-Interview),* as well as ‘*getting to know the person… what the person likes or doesn’t like, what the person can do, what they can’t do*’ *(Family carer 22-Interview).*

Valuing and respecting the client as ‘*a person with dementia. They’re not dementia*’ *(Homecare Worker 12-Interview)* was a skill that was imperative to developing positive relationships. Agencies could facilitate positive relationships by matching homecare workers with clients, based on age, similar interests or social and cultural values. In one example, we heard about a family discontinuing their support when the homecare worker’s care provision was perceived to not align with the client’s culture:

*‘The thing is, because she likes a certain way of cooking, a certain type of food, she didn’t want someone, you know, a Caucasian female coming in and making like a vegetable stew with water and some salt, and then here you are Madam. It’s not her cup of tea… So, it’s very difficult. She basically refused their assistance.’ (Family Carer 13-Interview)*

Developing relationships of familiarity often relied on consistent visit scheduling. Regularly seeing new faces was difficult for people living with dementia. Homecare workers also faced challenges when they were scheduled to visit a new client at short notice (i.e. following a hospital admission) or when allocated short visits. Clients living with dementia were understood to need more time, particularly if homecare workers were ‘*not as skilled as they could have been*’ *(Healthcare Professional 16-Interview)*.

### Subtheme 2: Managing complex attachments and boundaries

Complex mutual attachments formed, which could be difficult to navigate within the professional boundaries:

‘*I think we get quite attached to her as much as she’s got attached to us really… it’s like being with your gran. We’re not supposed to get emotionally involved, but I think we’re all human beings.*’ *(Homecare Worker 18-Interview)*

Both clients and homecare workers had ‘favourites’. Indeed, homecare workers described finding it hard to leave certain clients, often staying on in their own unpaid time. There was a sense in many narratives that work and contact with clients outside of contracted hours were virtuous and a sign of doing the job well:

‘*Sometimes, I can be there for the next half an hour, but I don’t care, just knowing that I’m doing something good. So, I try to make her as comfortable as possible because she’s on her own.*’ *(Homecare Worker 10-Interview)*

We observed situations where homecare workers brought flowers, food and home-baked goods for clients. In one situation, a homecare worker took a client’s family carer out for lunch in her own time. Becoming emotionally attached to clients and their family members could give rise to difficult emotions, particularly when clients died:

‘*I have to deal with then, the deaths of clients and it hits my staff really, really hard… I go to funerals of clients and people say sometimes you get hardened to things, but you don’t.*’ *(Homecare Manager 7-Interview)*

In one case, a homecare worker reported ‘dreading’ the loss of her favourite client but did not know of any support to help her deal with this.

## Managing when clients resist care

A key challenge facing homecare workers in supporting people living with dementia was how to work safely and effectively when clients displayed resistant behaviour. This often occurred during personal care. We observed a situation where a client was frustrated at being told she must stay in bed (a decision made by social workers and family due to deteriorated mobility); the homecare worker appeared unsure how to respond:

*‘Beatrice remains very distressed and shouts to be left alone. Audrey appears deflated but offers Beatrice a drink again; she pushes the cup away. Audrey comes over to me to apologise, saying “it isn’t very nice when Beatrice is like this”. Beatrice’s distress has been ongoing for 25-minutes and she remains asking to go to the toilet. Audrey has stopped responding.’ (Agency 4-Observation)*

With the same client, Audrey was observed to be ‘*physically keeping Beatrice on the bed with her own body*’, to prevent Beatrice from falling out of the bed as her frustration increased. Homecare workers and managers described training and support as important to equip homecare workers with the skills to manage such challenges, including where clients were verbally or physically aggressive:

‘*… [for someone] to be at the end of the phone, to say* “*look, I need somebody else here, this is getting a bit out of hand*”*. Then the support would have to be there, wouldn’t it? You can’t just put somebody in a situation with no armour, as such… You’ve been thrown to the wolves, haven’t you? You’ve got no training, how are you supposed to deal with somebody, with an illness that you know nothing about?*’ *(Homecare Worker 19-Interview)*

Understanding resistant behaviour as communication was described as important by all stakeholders; this was facilitated via familiarity and empathy:

‘*They might have toothache… When they start lashing out and becoming… Oh people say* “*well they’re a difficult person*”*. No, it’s probably because they’ve been in pain for a long time and besides, they’re also out of their mind from the pain and they’re tired from it, and they get angry.*’ *(Family carer 22-Interview)*

Experiential learning could enable homecare workers to develop these valuable skills. One homecare worker discussed encouraging others to try being moved in a hoist during training, to experience what it felt like. Ethical considerations also emerged when faced with client’s refusal; homecare workers required skill and knowledge to navigate between clients’ capacity and best interests.

## Drawing on agency and team support

Homecare agencies and therefore managers were crucial in providing emotional (subtheme 1) and practical (subtheme 2) support to homecare workers.

### Subtheme 1: Emotional needs

Homecare workers often worked in isolation, with limited regular contact with other workers or the agency. They described feeling a lack of emotional support when challenges arose:

‘*I think it would be nice to have somebody… that if it got too much like it was with [Client], that they could understand how I felt that day… And afterwards, I cried all the way home….*’ *(Homecare Worker 19-Interview)*

In contrast, some homecare workers were scheduled to work together with clients. One homecare worker who worked as a team providing a 24-hour care package reported feeling ‘*lucky to have a good team around*’, describing her relationship with the other care workers as ‘*a close unit*’ and ‘*supportive*’ *(Homecare Worker 18-Interview).*

Some homecare workers sought informal peer support during joint shifts. This was often their only opportunity for peer support but could lead to homecare workers chatting or venting frustrations in front of clients. In some observation visits, homecare workers would vent to the researcher-observers:

‘*Alina and Ashley talk to each other while they work and laugh together. Belinda gets distressed again by this and shouts. Alina says again that they are not laughing at her and says sorry.*’ *(Agency 3-Observation)*

All participant groups acknowledged the often challenging nature of the role and the importance of support from managers. Supportive approaches included managers adopting an open-door policy and office staff being always contactable, particularly when visits occurred outside of business hours.

### Subtheme 2: Practical support

Poorly organised visit scheduling caused frustration for all participant groups. Short-staffing and visit-cramming led to homecare workers having little time for breaks, training or self-care:

*‘We did a Care Certificate and I’m pretty sure we did some dementia training... Sorry, I’m yet to do it.… I was working when it was on.’ (Homecare Worker 26-Interview)*

Agencies faced difficulties when homecare workers called in sick at short notice. We observed office staff continuously phoning or texting homecare workers to find cover, often disrupting and distressing care workers and their clients during visits:

‘*While Amy is washing Beth, the phone in her back-pocket rings and she answers the call. It is the agency asking her to cover a shift. Amy continues to wash Beth’s private areas as she speaks on the phone. Amy moves towels over Beth’s body to keep her warm. Beth is silent.*’ *(Agency 4-Observation)*

Homecare workers often felt pressured to take on extra work, sometimes working long days without breaks. Busy schedules affected reliability and punctuality causing frustration for people living with dementia and their family carers:

*‘I know how difficult it is for [homecare workers] to stick to a sensible timetable. But, I don’t want to be greeting visitors unless I’m half dead at 11 at night, when they should have been there at 10 in the morning.’ (Person living with dementia 16-Interview)*

Homecare workers were sometimes visibly tired. We observed a homecare worker fall asleep during a ‘sitting service’ (a longer duration respite visit) and another who badly cut herself while washing-up dishes.

## Discussion

All stakeholders acknowledged the importance of skills to get to know and understand their clients living with dementia, to acknowledge their social and cultural values, to treat them with respect and to provide flexible, person-centred care. We found that greater role clarity and understanding of how to manage key relationships within the professional boundaries were important aspects of training for homecare workers. We described how homecare workers adopted a range of role identities, often in parallel, which brought conflicting expectations that could require skilful and at times, challenging negotiations.

Boundaries sometimes blurred between developing professional relationships and getting excessively emotionally involved, with a sense that it was up to individual homecare workers to navigate often close and complex relationships with clients and their families. Our findings echoed those of Schneider et al. (2019), who found that ‘going the extra mile was deployed in the organisation to indicate the standard of dedication that was expected of care workers’. We found that homecare workers faced difficulties in providing safe and effective support with clients who displayed resistant behaviours particularly during personal care. Perhaps the most powerful language we heard to signify the importance of training and support was of feeling ‘thrown to the wolves’ without sufficient training. While homecare was sometimes perceived as teamwork, it was more often isolating and overwhelming, with a lack of opportunity for peer or managerial support, even after the death of a client.

### Valuing flexible, responsive, person-centred care

The UK Home Care Association called for ‘greater flexibility for homecare providers to innovate and shape care with the individual’ (UKHCA, 2015). Adopting a more flexible way of working may involve homecare workers drawing upon their understanding of, and familiarity with their clients living with dementia, in order to provide responsive, person-centred care, tailored around the individual and their needs. Considering how to do this within everyday constraints may be an important dimension for training, with homecare workers have opportunities to reflect as a team on dilemmas and tensions, while exploring potential solutions.

Our findings regarding the diversity and ambiguity of the homecare worker role reflect those across other care staff (D’Astous et al., 2017; Vassos et al., 2013); this may suggest a desire for a more flexible working style and care approach. The growing popularity of directly employed care workers (i.e. Personal Assistants) in England reflects this desire among clients, families and homecare workers. These workers value the variety of their work and opportunities to adjust to the client’s needs and their relationships (Woolham et al., 2019), beyond the constraints of a care plan.

### Relationship-focused care

We found that a relationship-focused care approach was central to homecare for people living with dementia. Getting to know clients’ physical, emotional, social and cultural needs was identified as pivotal in developing these relationships. The different role identities that homecare workers adopted in practice may influence the relationships they form with clients, family carers and other care professionals. Social and communication skills can be developed through training; however, there are key organisational practices beyond training, that may be necessary to harness homecare relationships. We identified consistent visit scheduling and a process of matching homecare workers and clients as potentially helpful practices. Where a matching process is not possible within existing practice, training can develop homecare workers’ empathy and ‘emotional intelligence’ (Schneider et al., 2019). Relationship-focused care approaches (Nolan et al., 2004; Schneider et al., 2019) can be drawn upon to equip homecare workers with the skills to value reciprocity, mutuality and empathy, while supporting homecare workers to securely navigate and manage these relationships within the professional boundaries of the role.

We identified the presence of often complex family dynamics as important to acknowledge in training (see also Manthorpe et al., (2020)). Where homecare workers valued opportunities for peer support and team-based learning, this indicates that training in small groups to discuss and reflect on real-life examples may be effective for problem-solving. Using real-life examples and problem-solving can be helpful in acknowledging the complexities of homecare work (Surr et al., 2017).

### Supporting homecare workers

We heard about and observed the emotional and practical challenges faced by homecare workers when providing care to clients living with dementia. Facilitating opportunities for peer support and managers adopting an ‘open-door’, accessible communication approach were seen as ways to support homecare workers’ emotional needs, while incorporating breaks and avoiding visit-cramming could support their practical needs. Homecare workers reported experiencing grief (as well as anticipatory grief) when clients died. Training can help to prepare homecare workers for the loss of clients and dealing with the difficult emotions that were sometimes associated with the role. Beyond training, homecare agencies can support staff by making practical arrangements, for example, by enabling staff to attend clients’ funerals (Yeh et al., 2018).

Our findings regarding the effects of emotional labour on care staff are not new (D’Astous et al., 2017; Franzosa et al., 2019; Schneider et al., 2019); however, role ambiguity, unrealistic expectations and care provision in the intimate setting of the home may exacerbate this. Working in clients’ homes, as opposed to hospital or residential settings, may neutralise power dynamics and allow for greater autonomy and opportunities for informal care and relationship development (Bolton & Wibberley, 2014); there may be scope within training to consider and reflect on the impact of providing care in clients’ homes. The home as a site of care is complex and warrants further attention (Leverton et al., 2021).

## Practice implications

Drawing upon a rights-based approach, policy strategies emphasise a drive towards improving quality of life and quality of care for people living with dementia (Cahill, 2020). Our findings add to a growing evidence-base around dementia-specific training and support in achieving good quality dementia care, in addition to good working conditions and recognition for homecare workers.

Our findings build on previous work (e.g. the BOUGH study by Schneider et al. (2019)) indicating that the relational aspects of homecare for people living with dementia (including the relational complexities between stakeholders) are an important dimension of dementia-specific training and support. A reflective, team-based system of training may be effective in facilitating a more flexible and responsive way of working, within a protective and supportive professional framework. Unrealistic role expectations can have a detrimental impact on care provision and homecare worker well-being (D’Astous et al., 2017); thus, homecare agencies may also benefit from developing a clear message from the start of the service, outlining its scope so to set and manage expectations for clients living with dementia and family carers.

We found that homecare workers faced ethical dilemmas when providing care to clients living with dementia who displayed resistant behaviours (i.e. during personal care). Developing a clear understanding of how to provide care that is in the client’s best interests while also respecting their wishes is a key area for training. Experiential learning may help to develop necessary skills to deliver safe and effective care, with empathy, dignity and respect (Kiosses et al., 2016).

Training and support interventions cannot be considered in isolation from the wider context of social care. Organisational practice-level changes may be necessary to fully implement change into practice; a collaborative, whole systems approach can help to achieve this (McPherson, 2020; McPherson & Abell, 2020). For example, continuity of care and consistent scheduling facilitate relationship development that is valued by all stakeholders, but they require sufficient funding, staffing stability and capacity. Care workers on zero-hour contracts experience employment instability and poor mental health (Ravalier et al., 2019; Skills for Care, 2018); training and support alone cannot compensate for financial instability. Beyond this, constraints in this sector are not uniform. Typically, privately funded clients receive longer care visits, and local authority funders may be less able to pay homecare workers to attend training (Atkinson et al., 2018).

The UK Home Care Association’s (UKHCA, 2015) call for more consistent training for the homecare workforce includes advanced accredited training qualifications to allow specialisation, such as dementia care. There is an argument to professionalise the social care workforce to tackle its endemic problems (Dromey & Hochlaf, 2018). Regulated working policies would improve quality of care for clients and greater working conditions, recognition and value for staff (Scales et al., 2017). Yet professionalisation of the sector is debated, with fears that pressure of registering and attaining qualifications will drive staff away from an already short-staffed workforce (Hayes et al., 2019).

While we conducted this study before the COVID-19 pandemic, it has shone light on the importance and necessity of homecare, particularly for people living with dementia and their family carers (Giebel et al., 2020). Our finding that homecare workers often felt they had little support, even when faced with the death of a client, is heightened in the current circumstances. As some envision a kinder post-pandemic society which values essential workers, our study is a timely reminder of how far we may be from a homecare workforce that feels sufficiently valued, supported and trained.

## Strengths and limitations

This is, to our knowledge, the largest ethnographic study to consider the skills, training and support needs of homecare workers who provide care to people living with dementia, from the perspectives of key stakeholders.

Rapid ethnographies have been criticised for jeopardising the richness of data (Pink & Morgan, 2013), though they are considered well-suited to the more immediate priorities and concerns of applied health and care services (Burgess-Allen & Owen-Smith, 2010; Vindrola-Padros & Vindrola-Padros, 2018). The quality of our study was enhanced through data triangulation, documentation of the researcher-observers’ reflective stance and use of ‘fair dealing’ to represent a range of perspectives (Mays & Pope, 2000).

## Conclusion

The value of homecare for people living with dementia necessitates a workforce equipped with high-quality dementia-specific training and support, that acknowledges the key relational aspects of the homecare role. Where a flexible, responsive, person-centred service is required, training can enable homecare workers to feel secure and supported in doing so within a professional framework. The well-being of homecare workers can be reinforced through ongoing practical and emotional support from agency managers and peers. Corresponding organisational practices are needed, taking into account the wider context of the care sector where poignant challenges such as low pay and poor staff retention exist.

## Supplemental Material

sj-pdf-1-dem-10.1177_14713012211023676 – Supplemental Material for ‘You can’t just put somebody in a situation with no armour’. An ethnographic exploration of the training and support needs of homecare workers caring for people living with dementia Click here for additional data file. Supplemental Material, sj-pdf-1-dem-10.1177_14713012211023676 for ‘You can’t just put somebody in a situation with no armour’. An ethnographic exploration of the training and support needs of homecare workers caring for people living with dementia by Monica Leverton, Alexandra Burton, Jules Beresford-Dent, Penny Rapaport, Jill Manthorpe, Hassan Mansour, Stefanny Guerra Ceballos, Murna Downs, Quincy Samus, Briony Dow, Kathryn Lord and Claudia Cooper in Dementia
