# Supplementary material for: ‘You can’t just put somebody in a situation with no armour’. An ethnographic exploration of the training and support needs of homecare workers caring for people living with dementia
Source: Dementia (London). 2021 Jun 10;20(8):2982–3005. doi: 10.1177/14713012211023676 (PMC8678657; doi:10.1177/14713012211023676)
Supplement: sj-pdf-1-dem-10.1177_14713012211023676 – Supplemental Material for ‘You can’t just put somebody in a situation with no armour’. An ethnographic exploration of the training and support needs of homecare workers caring for people living with dementia [file sj-pdf-1-dem-10.1177_14713012211023676.pdf]

Appendices

A. Semi-structured interview topic guide

**Participant topic guide**	**Questions related to training and support for homecare workers**
	'We want to develop some training for homecare workers with clients living with dementia…'
Family caregivers	○ What do you think you and the person you care for would find most useful?<br>○ What do you think this should include?<br>○ Is there anything you wouldn't want this to include or be like?
People living with dementia	○ What do you think this should include?<br>○ If you could, is there anything you would like to tell/teach/show homecare workers or care agency managers?
Homecare staff and Health & Social Care Professionals	○ What do you think you and the person you care for would find most useful?<br>○ What do you think this should include?<br>○ Is there anything you wouldn't want this to include or be like?<br>○ What might make it easier/ more difficult to implement training for homecare workers working with clients living with dementia?<br>○ What might make it easier/ more difficult for homecare workers to carry out their training in practice?

B. <u>Semi-structured participant observation guide</u>

Guidance: Areas of interest to keep in mind during the observation:

1. A practical overview of the visit

a) record the time at which the home carer arrives and leaves the client's home

b) who is present

c) the environment in which the care is being delivered including physical layout, decor and cleanliness

d) "atmosphere" including general feelings about tension, is it welcoming, comfortable etc

e) the tasks that are delivered

2. Interactions and responses of home carers with clients and others

a) interactions and responses between the home carer and the client with dementia

b) interactions and responses between the home carer and others who may be present

- *positive, negative and neutral interactions/responses*
- *support of independence/ choice/ autonomy where possible*
- *challenges to independence (e.g. symptoms of distress, refusal of care or risks) and responses to it where this occurs, and whether these strategies are effective in resolving distress and enabling necessary care to be given*
- *emotional responses, strategies and resources used (e.g. practical, social, spiritual)*

c) how client (including behaviour, language, ethnicity and culture), family carer, home carer and/or management and organisational factors impact on care provided

d) whether additional needs arise, either stated by the person with dementia, family carer or home carer and how these are managed.

e) references to client or family carer goals or priorities, how these emerge and how they are acknowledged or not.

f) general thoughts and feelings about the care being delivered and how the care provided enables or disables independence

g) how your presence as a non-participant observer may have influenced your observations
